# An Easy-to-Use Machine Learning Model to Predict the Prognosis of Patients With COVID-19: Retrospective Cohort Study

**DOI:** 10.2196/24225

**Published:** 2020-11-09

**Authors:** Hyung-Jun Kim, Deokjae Han, Jeong-Han Kim, Daehyun Kim, Beomman Ha, Woong Seog, Yeon-Kyeng Lee, Dosang Lim, Sung Ok Hong, Mi-Jin Park, JoonNyung Heo

**Affiliations:** 1 Division of Pulmonary and Critical Care Medicine Department of Internal Medicine Armed Forces Capital Hospital Seongnam Republic of Korea; 2 Division of Infectious Diseases Department of Internal Medicine Armed Forces Capital Hospital Seongnam Republic of Korea; 3 Department of Periodontology Armed Forces Capital Hospital Seongnam Republic of Korea; 4 The Armed Forces Medical Command Seongnam Republic of Korea; 5 Division of Chronic Disease Control Korea Center for Disease Control and Prevention Cheongju Republic of Korea

**Keywords:** COVID-19, machine learning, prognosis, SARS-CoV-2, severe acute respiratory syndrome coronavirus 2

## Abstract

**Background:**

Prioritizing patients in need of intensive care is necessary to reduce the mortality rate during the COVID-19 pandemic. Although several scoring methods have been introduced, many require laboratory or radiographic findings that are not always easily available.

**Objective:**

The purpose of this study was to develop a machine learning model that predicts the need for intensive care for patients with COVID-19 using easily obtainable characteristics—baseline demographics, comorbidities, and symptoms.

**Methods:**

A retrospective study was performed using a nationwide cohort in South Korea. Patients admitted to 100 hospitals from January 25, 2020, to June 3, 2020, were included. Patient information was collected retrospectively by the attending physicians in each hospital and uploaded to an online case report form. Variables that could be easily provided were extracted. The variables were age, sex, smoking history, body temperature, comorbidities, activities of daily living, and symptoms. The primary outcome was the need for intensive care, defined as admission to the intensive care unit, use of extracorporeal life support, mechanical ventilation, vasopressors, or death within 30 days of hospitalization. Patients admitted until March 20, 2020, were included in the derivation group to develop prediction models using an automated machine learning technique. The models were externally validated in patients admitted after March 21, 2020. The machine learning model with the best discrimination performance was selected and compared against the CURB-65 (confusion, urea, respiratory rate, blood pressure, and 65 years of age or older) score using the area under the receiver operating characteristic curve (AUC).

**Results:**

A total of 4787 patients were included in the analysis, of which 3294 were assigned to the derivation group and 1493 to the validation group. Among the 4787 patients, 460 (9.6%) patients needed intensive care. Of the 55 machine learning models developed, the XGBoost model revealed the highest discrimination performance. The AUC of the XGBoost model was 0.897 (95% CI 0.877-0.917) for the derivation group and 0.885 (95% CI 0.855-0.915) for the validation group. Both the AUCs were superior to those of CURB-65, which were 0.836 (95% CI 0.825-0.847) and 0.843 (95% CI 0.829-0.857), respectively.

**Conclusions:**

We developed a machine learning model comprising simple patient-provided characteristics, which can efficiently predict the need for intensive care among patients with COVID-19.

## Introduction

COVID-19 is an ongoing global pandemic caused by the severe acute respiratory syndrome virus 2 with over 26 million confirmed cases worldwide as of August 31, 2020 [1]. The virus is highly transmissible [2] and commonly causes symptoms of fever, cough, fatigue, and myalgia [3]. The mortality rate varies from 0.4 to 304.9 deaths per 1000 COVID-19 cases in the United States according to age group [4], while underlying comorbidities and sex are frequently reported as risk factors for a grave prognosis [5,6].

Other than patient factors, the availability of medical resources is also a major factor for higher risk of death by COVID-19 [7]. The reported case fatality rates are higher in areas with sudden upsurges of COVID-19 compared to other regions, even in the same country. In China, the mortality rates were higher in Hubei Province, in which the outbreak sparked, compared to other provinces [8]. In South Korea, the estimated risk of death was 20.8% to 25.9% in Daegu and Gyeongsangbuk-do, which were regions that experienced a sudden COVID-19 outbreak, while other areas had a risk of 1.7% [9]. Such findings are due to the availability of hospital beds, medical professionals, and other necessary supplies. Therefore, prioritizing patients in need of intensive care is crucial to prevent unnecessary consumption of medical resources by mild or asymptomatic patients.

There have been previous efforts to elucidate the risk factors of grave prognoses among patients with COVID-19 [10-13]. A previous report from China used patient demographics, symptoms, comorbidities, lactate dehydrogenase level, neutrophil–lymphocyte ratio, and radiographic abnormality to predict intensive care unit (ICU) admission, invasive ventilation, or death [10]. Another study from Italy concluded that the proportion of well-aerated lungs was associated with ICU admission or death [13]. Other studies from China also emphasized the use of laboratory findings to predict severe types of COVID-19 [11,12]. Although the performance of these models was excellent, they included laboratory or radiographic findings that may not be quickly available in underdeveloped areas. In addition, rapid adjustment of the scoring systems is not feasible when additional data are collected.

In this study, we aimed to develop a prediction model with information that can easily be provided by patients, limited to baseline demographics, comorbidities, and subjective symptoms. The model aimed to predict the need for intensive care among patients with COVID-19 using an automated machine learning (AutoML) technique [14], which can easily adjust the relative importance of different features as further data become available.

## Methods

### Data Source and Study Population

This was a retrospective study using a nationwide cohort that included all hospitalized patients with COVID-19 in South Korea, developed and managed by the Korean Centers for Disease Control and Prevention. Patients with laboratory-confirmed COVID-19 were either admitted to a hospital or a community treatment center. The Korean Centers for Disease Control and Prevention requested that all hospitals with patients with COVID-19 register and record their patients’ data to the cohort. Data were collected retrospectively through medical chart review by the attending physicians in each center, and were uploaded to an online case report form [15].

Among the patients with COVID-19 hospitalized from January 25, 2020, those who died or were released from quarantine as of June 3, 2020, were included in this study. Patients who were admitted until March 20 were assigned to the derivation group, and those hospitalized after March 21 were assigned to the temporal external validation group. The cut-off point of March 20 was arbitrary. However, two major changes occurred in clinical practice during the study period. First, as testing capacity increased during the pandemic, testing criteria were broadened after February 20. Second, services of community treatment centers commenced on March 2, which we used to quarantine patients with mild symptoms. We excluded patients aged <18 years and those with missing data. This study was approved by the Institutional Review Board of the Armed Forces Medical Command (approval number: AFMC-20053-IRB-20-053) with a waiver of informed consent due to the retrospective nature of the study.

### Variable Selection

Variables used for developing the machine learning model included information that could easily be provided by patients without the need for laboratory or radiographic evaluation. The variables were age, sex, smoking history, body temperature, underlying comorbidities, activities of daily living (ADL), and symptoms reported by the patients. Comorbidities included diabetes, heart failure, hypertension, asthma, chronic obstructive pulmonary disease, chronic kidney disease, cancer, chronic liver disease, chronic neurological disorders, chronic hematologic disorders, HIV infection, autoimmune diseases, dementia, and pregnancy. The ADL scale was divided into three categories: independent, partially dependent, and totally dependent. Symptoms considered in the cohort were mental status, cough, sputum, hemoptysis, sore throat, rhinorrhea, chest discomfort, myalgia, arthralgia, fatigue, dyspnea, anosmia, headache, vomiting, and diarrhea.

The CURB-65 score, which stands for confusion, urea, respiratory rate, blood pressure, and 65 years of age or older, was chosen as a comparison against the machine learning model [16]. The score consists of mentality, blood urea nitrogen level, respiratory rate, blood pressure, and age [16]. These data were also extracted from the cohort. Levels of blood urea nitrogen were extracted only to calculate the CURB-65 score and were not included in the machine learning model.

### Outcome for the Prediction Models

The primary outcome was predicting need for intensive care, which we defined as admission to the ICU, use of extracorporeal life support, mechanical ventilation, vasopressors, or death during the first 30 days of admission. Information on the use of extracorporeal life support, mechanical ventilation, or vasopressors was included to account for patients who could not be admitted to the ICU due to limited availability.

### Machine Learning Analysis

Complete case analysis was performed, and continuous variables were inspected for input errors. AutoML was used to automate the process of constructing pipelines for the development of the machine learning models, such as hyperparameter optimization and model training. H2O.ai was used to develop these AutoML models [14,17]. 

The algorithms used during the development of the prediction models using AutoML can be classified into three categories: linear, decision tree based, and neural network based. Linear algorithms are essentially multidimensional linear mathematical formulas. They are intuitive and easy to interpret, and problems that can be described in a linear manner would be best solved by these algorithms. Decision tree–based algorithms consist of a multitude of decision trees comprising multiple true or false conditions for input variables. We used the sum of the decisions made by the decision trees for final classification. These models are better for processing categorical variables with multiple levels, and they can account for interactions between variables. A neural network comprises layers of interconnected artificial neurons that are designed based on a biological neuron. These artificial neurons receive multiple inputs that are multiplied by weights, and they output the sum of these inputs. Neural network models are difficult to interpret, but they can successfully represent complicated interactions between inputs. However, these models are not ideal for representing categorical inputs with multiple levels. Since it is unclear which algorithm can best explain the current problem, all these algorithms were used to develop predictive models, which were then compared based on their discriminative power.

The following models were trained in the AutoML process: 3 prespecified XGBoost gradient boosting machine models, a fixed grid of generalized linear models, a default random forest, 5 prespecified H2O gradient boosting machines, a near-default deep neural network, an extremely randomized forest, a random grid of XGBoost gradient boosting machines, a random grid of H2O gradient boosting machines, and a random grid of deep neural network models. Two stacked ensemble models were developed using the aforementioned developed models [18].

### Other Statistical Considerations

Descriptive statistics were performed for all variables in both derivation and validation groups. Patient characteristics were summarized as counts with proportions for categorical variables and median with interquartile range for continuous variables. Results of the calculated probability based on the machine learning model have been presented in numbers ranging from 0 to 100, with 0 being the lowest probability of requiring intensive care, and 100 being the highest. The numbers were used to calculate area under the receiver operating characteristic curve (AUC) in the derivation and validation groups. For the derivation group, the mean value of the AUC for the 5 cross-validation sets of each model was used to compare the performance of the developed models. Receiver operating characteristic (ROC) curves were drawn, and the areas under the curves were calculated to assess the predictive performance of the models. *P* were calculated between the AUC of the machine learning model and the CURB-65 score. Sensitivity, specificity, positive predictive value (PPV), negative predictive value (NPV), and F-measures were measured for different cut-off values. Confusion matrices were constructed for both derivation and validation groups. All *P* values were two-sided, and a *P* value of <.05 was considered statistically significant. Statistical analysis was performed using R 4.0.0 (The R Foundation), with the pROC package to draw the ROC curves [19].

## Results

### Patient Characteristics

**Figure 1:**
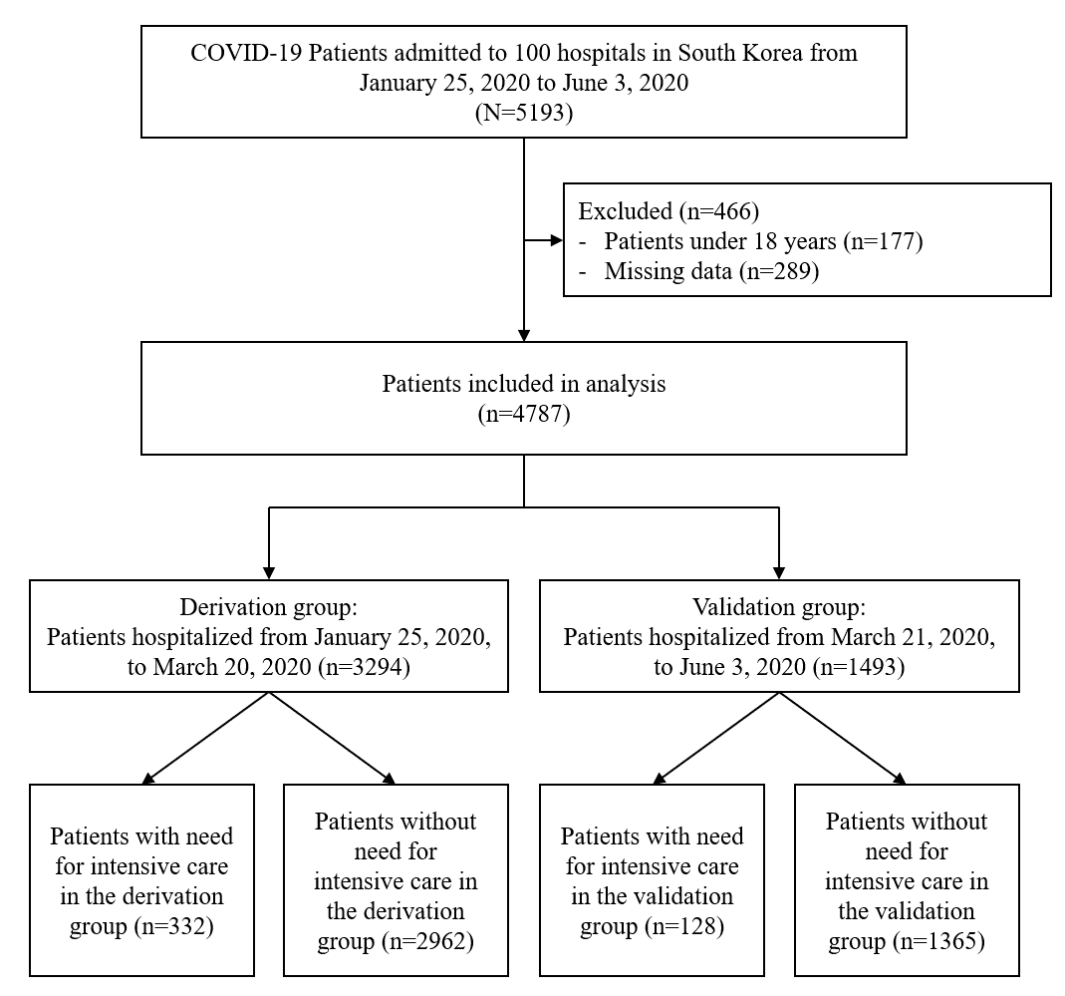
Flowchart of the patient selection process. Patients hospitalized in 100 hospitals in South Korea from January 25, 2020, to June 3, 2020, were included. Patients who were admitted until March 20 were assigned to the derivation group, and those hospitalized after March 21 were assigned to the validation group.

A total of 5193 patients with polymerase chain reaction–confirmed COVID-19 from 100 centers were registered with the nationwide cohort during the study period. Patients under 18 years (n=117, 2.2%) and those with missing data (n=289, 5.6%) were excluded, leaving 4787 patients for analysis. Among these patients, 3294 were assigned to the derivation group, and the remaining 1493 patients were assigned to the validation group (Figure 1).

Compared to the patients in the validation group, those in the derivation group were older (median 57.0 years [IQR 42.0-68.0] vs 53.0 years [IQR 30.0-66.0]; *P*<.001), with a lower proportion of males (n=1227, 37.2% vs n=681, 45.6%; *P*<.001). Patients in the derivation group experienced more symptoms, including cough (n=1537, 46.6% vs n=440, 29.5%; *P*<.001), sputum (n=1054, 32.0% vs n=304, 20.4%; *P*<.001), headache (n=599, 18.2% vs n=165, 11.1%; *P*<.001), and myalgia (n=568, 17.2% vs n=159, 10.7%; *P*<.001), but less anosmia (n=40, 1.2% vs n=97, 6.5%; *P*<.001) compared to the validation group. The presence of underlying comorbidities was largely similar between the two groups, except for hypertension (n=883, 26.8% vs n=351, 23.5%; *P*=.02) and diabetes (n=537, 16.3% vs n=204, 13.7%; *P*=.02), which were more common in the derivation group, and dementia (n=182, 5.5% vs n=153, 10.3%; *P*<.001), which was more common in the validation group. Patients in the derivation group were more independent in terms of their ADL compared to those in the validation group (n=2932, 89.0% vs n=1188, 79.6%; *P*<.001).

A total of 460 patients (9.6%) suffered the need for intensive care, of which 221 (4.6%) patients were admitted to the ICU, and 223 (4.7%) died (Table 1).

**Table 1:** Descriptive statistics for the included patients according to derivation and validation groups.

Variable		Total patients (N=4787)	Derivation group (n=3294)	Validation group (n=1493)	*P* value
Age (years), median (IQR)		55.0 (38.0-68.0)	57.0 (42.0-68.0)	53.0 (30.0-66.0)	<.001
Sex (male), n (%)		1908 (39.9)	1227 (37.2)	681 (45.6)	<.001
**Smoking history, n (%)**					<.001
	Never smoked	4388 (91.7)	3084 (93.6)	1304 (87.4)	N/A^a^
	Former smoker	136 (2.8)	97 (2.9)	39 (2.6)	N/A
	Current smoker	263 (5.5)	114 (3.5)	149 (10.0)	N/A
Body temperature (℃), median (IQR)		36.8 (36.5-37.2)	36.9 (36.5-37.3)	36.8 (36.5-37.2)	.002
**Symptoms, n (%)**					
	Cough	1977 (41.3)	1537 (46.6)	440 (29.5)	<.001
	Sputum	1358 (28.4)	1054 (32.0)	304 (20.4)	<.001
	Headache	764 (16.0)	599 (18.2)	165 (11.1)	<.001
	Myalgia	727 (15.2)	568 (17.2)	159 (10.7)	<.001
	Sore throat	688 (14.4)	513 (15.6)	175 (11.7)	.001
	Dyspnea	654 (13.7)	543 (16.5)	111 (7.4)	<.001
	Rhinorrhea	424 (8.9)	318 (9.7)	106 (7.1)	.005
	Diarrhea	399 (8.3)	327 (9.9)	72 (4.8)	<.001
	Chest pain	369 (7.7)	305 (9.3)	64 (4.3)	<.001
	Nausea/vomiting	225 (4.7)	176 (5.3)	49 (3.3)	.002
	Fatigue	188 (3.9)	149 (4.5)	39 (2.6)	.002
	Anosmia	137 (2.9)	40 (1.2)	97 (6.5)	<.001
	Hemoptysis	26 (0.5)	23 (0.7)	3 (0.2)	.051
	Altered mentality	37 (0.8)	22 (0.7)	15 (1.0)	.29
	Arthralgia	18 (0.4)	16 (0.5)	2 (0.1)	.11
**Comorbidities, n (%)**					
	Hypertension	1234 (25.8)	883 (26.8)	351 (23.5)	.02
	Diabetes	741 (15.5)	537 (16.3)	204 (13.7)	.02
	Dementia	335 (7.0)	182 (5.5)	153 (10.3)	<.001
	Chronic cardiac disease	195 (4.1)	142 (4.3)	53 (3.6)	.25
	Cancer	160 (3.3)	113 (3.4)	47 (3.2)	.68
	Asthma	123 (2.6)	95 (2.9)	28 (1.9)	.052
	Chronic liver disease	80 (1.7)	56 (1.7)	24 (1.6)	.92
	Heart failure	70 (1.5)	44 (1.3)	26 (1.7)	.34
	Chronic kidney disease	60 (1.3)	48 (1.5)	12 (0.8)	.08
	Chronic obstructive pulmonary disease	42 (0.9)	35 (1.1)	7 (0.5)	.06
	Chronic neurologic disorder	42 (0.9)	24 (0.7)	18 (1.2)	.14
	Chronic hematologic disorder	35 (0.7)	28 (0.8)	7 (0.5)	.21
	Autoimmune disease	34 (0.7)	27 (0.8)	7 (0.5)	.25
	Pregnancy	20 (0.4)	13 (0.4)	7 (0.5)	.90
	HIV infection	10 (0.2)	7 (0.2)	3 (0.2)	>.99
**Activities of daily living, n (%)**					<.001
	Independent	4120 (86.1)	2932 (89.0)	1188 (79.6)	N/A
	Partially dependent	375 (7.8)	203 (6.2)	172 (11.5)	N/A
	Totally dependent	292 (6.1)	160 (4.9)	132 (8.8)	N/A
**Need for intensive care, n (%)**		460 (9.6)	332 (10.1)	128 (8.6)	.12
	Death	223 (4.7)	161 (4.9)	62 (4.2)	.30
	Admission to ICU^b^	221 (4.6)	169 (5.1)	52 (3.5)	.02
	Vasopressor treatment	119 (2.5)	84 (2.5)	35 (2.3)	.75
	Mechanical ventilation	66 (1.4)	54 (1.6)	12 (0.8)	.001
	Extracorporeal life support	27 (0.6)	21 (0.6)	6 (0.4)	.43

^a^N/A: not applicable.

^b^ICU: intensive care unit.

### Derivation and Internal Validation of the Machine Learning Model

**Figure 2:**
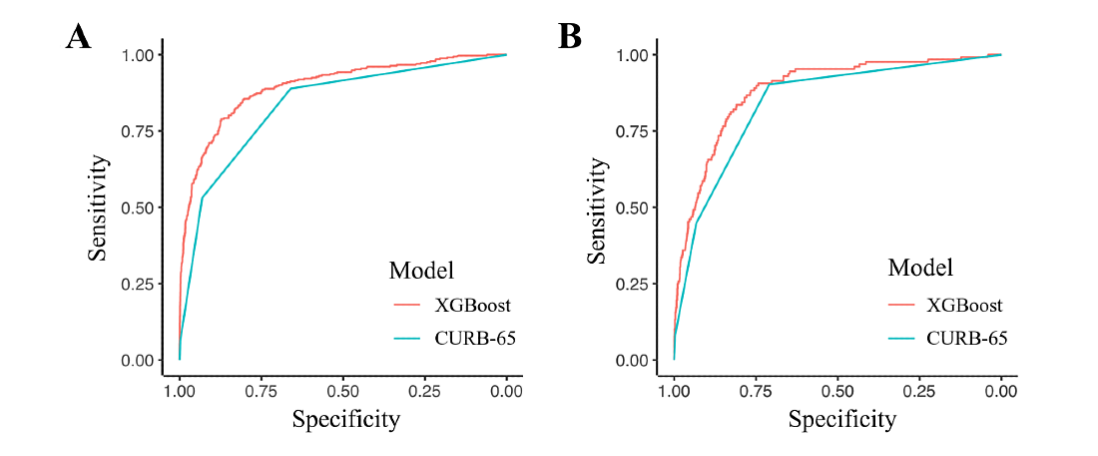
Receiver operating characteristic curves for the machine learning model (XGBoost) and the CURB-65 (confusion, urea, respiratory rate, blood pressure, and 65 years of age or older) score for predicting patients requiring intensive care. (A) Comparison in the derivation group, where the area under the receiver operating characteristic (AUC) curves were 0.897 for the gradient boosting machine model, and 0.836 for the CURB-65 score (*P*<.001). (B) Comparison in the temporal external validation group, where the AUC were 0.885 for the machine learning model, and 0.843 for the CURB-65 score (*P*=.01).

With the AutoML, 55 machine learning models were developed to predict the need for intensive care among patients with COVID-19. The XGBoost model, which showed an AUC of 0.897 (95% CI 0.877-0.917) by cross-validation in the derivation group, was chosen as the best machine learning model (Multimedia Appendix 1). The important features of this model were ADL, age, dyspnea, initial body temperature, sex, and underlying comorbidities. More detailed information on each feature is presented in Multimedia Appendix 2. The developed machine learning model revealed significantly better discrimination performance than the CURB-65 score (AUC 0.836 with 95% CI 0.825-0.847, *P*<.001) for predicting the need for intensive care among patients with COVID-19. A comparison of the ROC curves for the XGBoost machine learning model and the CURB-65 score is shown in Figure 2A.

### External Validation of the Model

External validation was performed in the validation group, using the developed XGBoost machine learning model. The discrimination performance of the machine learning model showed an AUC of 0.885 with 95% CI 0.855-0.915, which was significantly higher than that of CURB-65 (0.843, 95% CI 0.829-0.857, *P*=*.*01) (Figure 2B).

### Comparison of the Machine Learning Model With the CURB-65 Score With Different Thresholds

With a cut-off value of 0.5, the CURB-65 score showed a sensitivity of 0.89, specificity of 0.66, PPV of 0.05, NPV of 1.00, and F-measure of 0.10. A cut-off value of 0.06 for the XGBoost machine learning model, which shows a similar sensitivity (0.89), revealed a specificity of 0.75, PPV of 0.36, NPV of 0.99, and F-measure of 0.43. The XGBoost score also revealed better specificity, PPV, and F-measure compared to CURB-65 when different cut-off thresholds were used (Table 2). The confusion matrices of the developed model for the development and validation groups are shown in Multimedia Appendix 3.

**Table 2:** Sensitivity, specificity, positive predictive value (PPV), negative predictive value (NPV), and F-measure for the machine learning model and the CURB-65 (confusion, urea, respiratory rate, blood pressure, and 65 years of age or older) score, with different cut-offs.

Cut-off	Sensitivity	Specificity	PPV	NPV	F-measure
CURB-65 score >0.5	0.89	0.66	0.05	1.00	0.10
XGBoost score >0.06	0.89	0.75	0.36	0.99	0.43
CURB-65 score >1.5	0.53	0.93	0.14	0.99	0.22
XGBoost score >0.34	0.53	0.97	0.63	0.95	0.58
CURB-65 score >2.5	0.06	1.00	0.40	0.98	0.11
XGBoost score >0.89	0.06	1.00	0.95	0.90	0.12

### Web Application of Prediction Models

**Figure 3:**
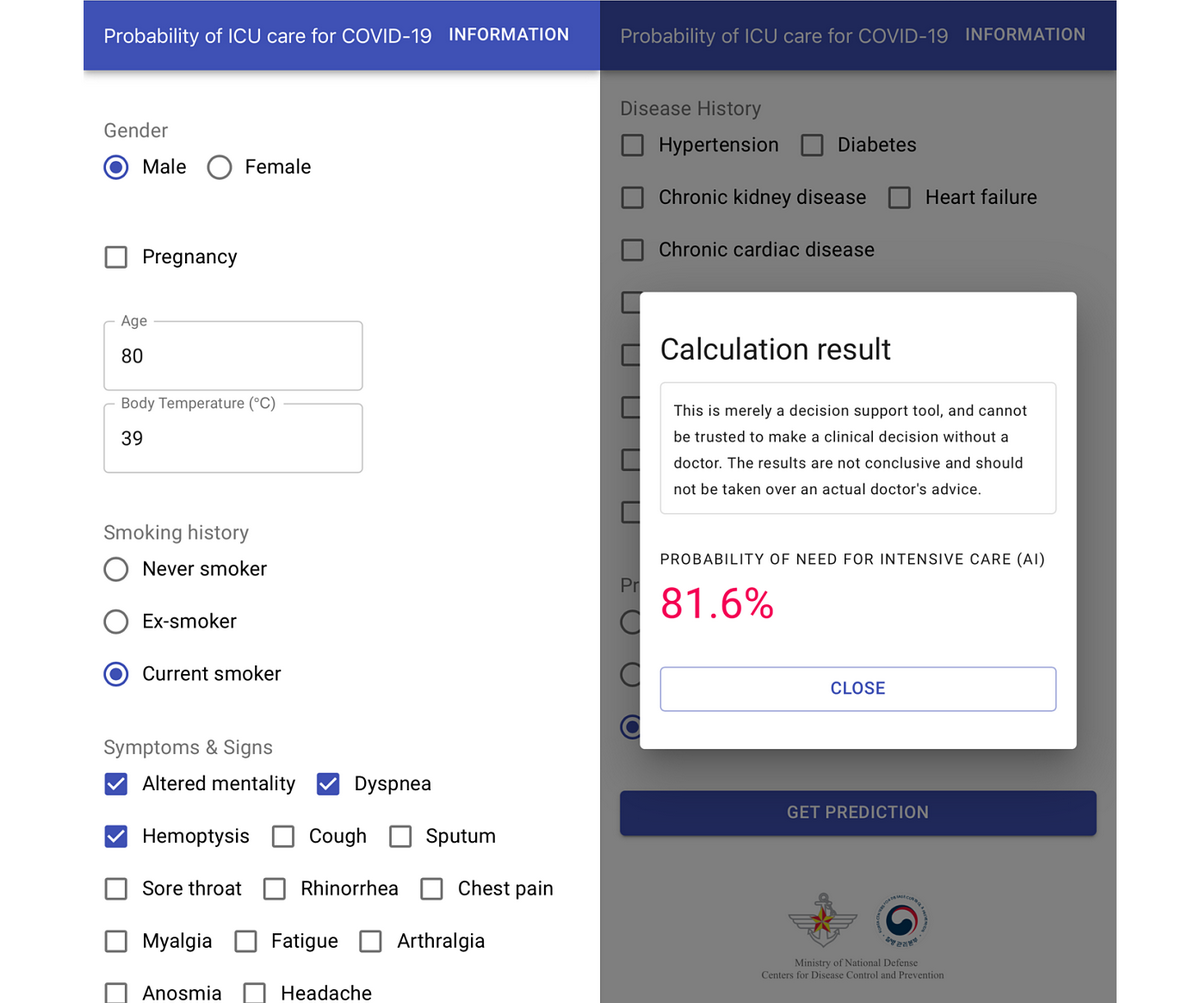
Screenshots of the web-based application for easy usage of the developed machine learning model [20]. After input of simple patient-derived information, the probability of the need for intensive care within 30 days is calculated.

A web-based application was developed for better accessibility and easy use of the models. The application can be accessed online [20] (Figure 3), and it has been enlisted in the World Health Organization’s Digital Health Atlas [21]. The application calculates the probability of need for intensive care, which is computed according to the derived model. However, it does not store any data yet. It is intended for use by medical practitioners to aid with medical decisions.

## Discussion

### Principal Results

This study presents a machine learning model that predicts the need for intensive care among patients with COVID-19 from a nationwide cohort in South Korea, including 100 hospitals. The model was derived from the data of patients who were hospitalized from January 25, 2020, to March 20, 2020, and was validated in a separate group of patients hospitalized between March 21, 2020, and June 3, 2020. The AUC of the machine learning model was 0.897 (95% CI 0.877-0.917) for the derivation cohort and 0.885 (95% CI 0.855-0.915) for the validation cohort, which revealed better discrimination performance than that of CURB-65. Important features included ADL, age, dyspnea, initial body temperature, and sex.

### Comparison With Prior Work

The main features selected in the machine learning model are mostly coherent with previous reports. Older age and male sex have been constantly emphasized as major risk factors for adverse outcomes in patients with COVID-19 [6,22,23]. An early report on 85 fatal cases of COVID-19 in Wuhan [24] revealed that the mean age of patients was 65.8 years, and 62 of the 85 patients (72.9%) were male [5]. Dyspnea was also a major factor in our study. Incidence of dyspnea is relatively low in COVID-19 as compared to other respiratory symptoms, despite the common pneumonic infiltration on chest radiographs [25]. In a recent systematic review that included 43 studies [3], shortness of breath was observed in 49.2% in patients with critical illness, while bilateral pneumonia was observed in chest computed tomography (CT) images of 91.0% of patients with the same disease extent. In an earlier study in China, even in severely ill patients, dyspnea was observed in about 37.6% of the patients [26]. Therefore, the presentation of dyspnea may imply extensive involvement of the lungs, which leads to grave prognoses [5]. Underlying comorbidities were also repeatedly highlighted as major risk factors for poor prognoses of patients with COVID-19. A pooled analysis of COVID-19 reports emphasized that hypertension is associated with an approximately 2.5-fold increased risk of higher severity and mortality [27]. Another previous study of 174 patients revealed that patients with diabetes were at higher risk of pneumonia, release of tissue injury-related enzymes, and higher rates of inflammatory responses [28]. Such findings are well summarized in a systematic review that included 3027 patients [5]: male sex (pooled odds ratio [OR] 1.76 with 95% CI 1.41-2.18), age over 65 years (pooled OR 6.06 with 95% CI 3.98-9.22), dyspnea (pooled OR 4.16 with 95% CI 3.13-5.53), presence of cardiovascular disease (pooled OR 5.19 with 95% CI 3.25-8.29), diabetes (pooled OR 3.68 with 95% CI 2.68-5.03), and hypertension (pooled OR 2.72 with 95% CI 1.60-4.64) were all significant factors associated with the progression of COVID-19.

In addition to previous reports, ADL limitation and abnormal body temperature were associated with the need for intensive care among patients with COVID-19 in our study. ADL limitation is known to be an independent risk factor for mortality among elderly patients with pneumonia [29,30]. Because most of the poor outcomes occur in the elderly in COVID-19 [6,22,23], it is probable that ADL limitation leads to the need for intensive care. Abnormal body temperature is also a well-known risk factor for grave prognosis in community-acquired pneumonia patients [31].

### Strengths of This Study

Our machine learning prediction model based on simple patient demographics and subjective symptoms can be useful for the early triage of patients in this pandemic situation. First, it uses information that can be easily provided without advanced equipment, such as age, sex, past medical history, and subjective symptoms. Previous scoring systems [10,11,32-35], including a recently reported deep learning model [36], require laboratory or radiographic findings as the main variables. Although such models can be helpful in fully equipped medical facilities, they initially consume a certain amount of medical resources and time. In areas where laboratory exams or CT exams are limited, our scoring model can be an effective solution for earlier triage. Second, because our model is based on the AutoML technique [20], the relative importance of the features can easily be adjusted with the newly acquired patient data. AutoML techniques have been studied extensively [14] and are expected to be useful for many applications, including in the field of health care [17]. AutoML mainly helps in building machine learning pipelines, which requires expertise in machine learning and is time consuming. It is effective when the time or resources necessary for building a high-functioning model are limited. Considering the rapid adaptability of our model, it can be used effectively with populations with different ethnic or regional backgrounds when further data are collected from the similar populations. It is useful in this pandemic situation, where insufficiency of medical resources has been identified as a critical factor in patient survival [7]. Especially in contexts with less than adequate medical staff, the web-based application is easy to use owing to its intuitive interfaces and clear guides, making it possible for the attending physicians to triage patients without adequate medical knowledge about COVID-19.

CURB-65 was used for comparison with the AutoML model in our study. CURB-65 is a well-known score derived and validated for predicting mortality among patients with community-acquired pneumonia [16], and also shows promising performance in patients with COVID-19 [37]. It is comprised of 6 variables: mental status, levels of blood urea nitrogen, respiratory rate, blood pressure, and age. COVID-19 commonly accompanies pneumonia [4]. In a recent systematic review [38], bilateral (72.9%) or unilateral (25.0%) involvement of chest X-rays was observed among patients with confirmed COVID-19. A large proportion of the involvement is ground-glass opacities (68.5%), which are difficult to recognize from simple chest radiographs. In our study, 2050 of the 4787 patients (42.8%) underwent chest CT evaluation, and among them, 1535 (74.9%) were recognized to have pneumonic infiltrations.

### Recommendations

Our model can be used as a decision-support system for medical professionals when active monitoring is not possible due to patient overload caused by the lack of availability of medical staff. However, we cannot recommend a uniform cut-off value for patient transfer to higher-level facilities because this decision depends on the local situation. This decision needs to be made considering the availability of beds in higher-level facilities, the rate of regional increase in the number of patients with COVID-19, and the treatment capability of the facility the patient is currently admitted to. Yet, one solid recommendation that can be made is to prioritize the transfer of patients with a higher probability of need for intensive care when feasible.

### Limitations

Our study had several limitations. First, the sample excluded patients assigned to community treatment centers. However, assignment to community treatment centers was mostly conducted for quarantining purposes, not for active treatment. When they required active treatment, such patients were transferred to hospitals and were eventually included in this study. Second, our data set was imbalanced, with 9.6% of the patients requiring intensive care. Third, our initial model was built based on patients from South Korea. Nevertheless, due to the nature of AutoML, the model can be updated easily when further data become available.

### Conclusions

In conclusion, we derived and validated a machine learning prediction model comprising simple patient-provided characteristics. The model included variables that were largely consistent with previous reports, and it can efficiently anticipate deterioration among patients with COVID-19. The model is easy to use and adjust, requires minimal resources, and can be an effective solution for easy triage in areas with a shortage of medical resources. The model can be used for patient monitoring, and also has a potential as a warning system for self-quarantined patients. However, in the future, randomized trials need to be conducted to examine the direct impact of our model on patient survival.

## Appendix

Multimedia Appendix 1 AUROC curves of the developed machine learning models.

Multimedia Appendix 2 Feature importance of the gradient boosting machine model for prediction of patients requiring intensive care.

Multimedia Appendix 3 Confusion matrices for the development and validation groups.

## Abbreviations

ADL: activities of daily living
AUC: area under the receiver operating characteristic curve
AutoML: automated machine learning
CT: computed tomography
CURB-65: confusion, urea, respiratory rate, blood pressure, and 65 years of age or older
ICU: intensive care unit
NPV: negative predictive value
OR: odds ratio
PPV: positive predictive value
ROC: receiver operating characteristic
