# Supplementary material for: An Easy-to-Use Machine Learning Model to Predict the Prognosis of Patients With COVID-19: Retrospective Cohort Study
Source: J Med Internet Res. 2020 Nov 9;22(11):e24225. doi: 10.2196/24225 (PMC7655730; doi:10.2196/24225)
Supplement: Multimedia Appendix 1 [file jmir_v22i11e24225_app1.docx]

**Multimedia Appendix 1. Area under the receiver operating characteristic curves of the developed machine learning models.**

| **Model algorithm** | **Model Number** | **AUC (Cross validation) ^a^** | **AUC (Training)** |
| --- | --- | --- | --- |
| XGBoost with grid search | 26 | 0.861 | 0.897 |
| XGBoost with grid search | 22 | 0.861 | 0.902 |
| XGBoost with grid search | 5 | 0.861 | 0.901 |
| Generalized linear model | 1 | 0.860 | 0.873 |
| XGBoost with grid search | 14 | 0.859 | 0.893 |
| GBM with grid search | 5 | 0.858 | 0.889 |
| GBM | 2 | 0.853 | 0.944 |
| XGBoost with grid search | 24 | 0.853 | 0.881 |
| GBM with grid search | 3 | 0.853 | 0.875 |
| XGBoost with grid search | 10 | 0.852 | 0.870 |
| XGBoost with grid search | 29 | 0.852 | 0.883 |
| GBM | 3 | 0.852 | 0.953 |
| GBM | 4 | 0.851 | 0.971 |
| XGBoost with grid search | 7 | 0.850 | 0.889 |
| XGBoost with grid search | 18 | 0.850 | 0.880 |
| GBM with grid search | 9 | 0.849 | 0.915 |
| GBM | 1 | 0.849 | 0.943 |
| XGBoost with grid search | 11 | 0.849 | 0.886 |
| DRF | 1 | 0.849 | 0.824 |
| GBM with grid search | 11 | 0.849 | 0.876 |
| XGBoost with grid search | 27 | 0.848 | 0.878 |
| GBM | 5 | 0.848 | 0.912 |
| XGBoost with grid search | 6 | 0.846 | 0.951 |
| XGBoost with grid search | 13 | 0.846 | 0.891 |
| GBM with grid search | 6 | 0.845 | 0.930 |
| GBM with grid search | 4 | 0.845 | 0.903 |
| XGBoost with grid search | 19 | 0.844 | 0.938 |
| GBM with grid search | 2 | 0.843 | 0.979 |
| XGBoost with grid search | 3 | 0.843 | 0.891 |
| XGBoost with grid search | 8 | 0.843 | 0.902 |
| XGBoost | 2 | 0.842 | 0.896 |
| XRT | 1 | 0.842 | 0.847 |
| XGBoost with grid search | 16 | 0.842 | 0.904 |
| XGBoost with grid search | 12 | 0.841 | 0.916 |
| XGBoost | 3 | 0.841 | 0.930 |
| XGBoost with grid search | 20 | 0.840 | 0.916 |
| XGBoost | 1 | 0.837 | 0.921 |
| XGBoost with grid search | 4 | 0.837 | 0.921 |
| GBM with grid search | 10 | 0.835 | 0.972 |
| GBM with grid search | 1 | 0.834 | 0.978 |
| XGBoost with grid search | 28 | 0.830 | 0.955 |
| XGBoost with grid search | 1 | 0.828 | 0.992 |
| XGBoost with grid search | 23 | 0.828 | 1.000 |
| XGBoost with grid search | 2 | 0.826 | 0.997 |
| XGBoost with grid search | 21 | 0.816 | 0.999 |
| XGBoost with grid search | 25 | 0.815 | 1.000 |
| XGBoost with grid search | 15 | 0.814 | 1.000 |
| XGBoost with grid search | 9 | 0.813 | 0.992 |
| XGBoost with grid search | 17 | 0.809 | 1.000 |
| GBM with grid search | 8 | 0.801 | 1.000 |
| GBM with grid search | 7 | 0.788 | 1.000 |
| Deep Learning with grid search | 3 | 0.736 | 0.964 |
| Deep Learning with grid search | 2 | 0.684 | 0.901 |
| Deep Learning with grid search | 1 | 0.681 | 0.957 |
| Deep Learning | 1 | 0.645 | 0.876 |

AUC, area under the receiver operating characteristic curve; GBM, gradient boosting machine; DRF, distributed random forest; XRT, extremely randomized trees.

^a^The mean of AUCs of the five cross validation results.
