# Supplementary material for: An Easy-to-Use Machine Learning Model to Predict the Prognosis of Patients With COVID-19: Retrospective Cohort Study
Source: J Med Internet Res. 2020 Nov 9;22(11):e24225. doi: 10.2196/24225 (PMC7655730; doi:10.2196/24225)
Supplement: Multimedia Appendix 2 [file jmir_v22i11e24225_app2.docx]

**Multimedia Appendix 2. Feature importance of the gradient boosting machine model for prediction of patients requiring intensive care.**

| **Feature** | **Relative importance** |
| --- | --- |
| ADL score 0 | 238.6871 |
| Age | 226.3849 |
| No dyspnea | 187.6901 |
| Initial body temperature | 87.8135 |
| ADL score 2 | 67.0419 |
| Female | 33.1858 |
| Dyspnea | 29.2905 |
| Male | 18.3267 |
| No chronic kidney disease | 18.2495 |
| Normal mentality | 11.6689 |
| Ex-smoker | 9.7603 |
| No headache | 9.5735 |
| No dementia | 9.0295 |
| No chronic cardiac disease | 8.0522 |
| No hemoptysis | 7.68 |
| Not fatigued | 6.4137 |
| No hypertension | 6.3221 |
| Hypertension | 6.2688 |
| Heart failure | 5.9429 |
| Diabetes | 5.2309 |
| Current smoker | 5.066 |
| No sore throat | 4.5632 |
| Dementia | 4.3034 |
| Chronic kidney disease | 4.2617 |
| No heart failure | 4.1133 |
| No cancer | 3.9166 |
| No chronic hematologic disorder | 3.7485 |
| No COPD | 3.7446 |
| Headache | 3.661 |
| Chronic hematologic disorder | 3.6492 |
| No nausea or vomiting | 3.5416 |
| No chest pain | 3.459 |
| Sore throat | 3.3471 |
| No cough | 3.2329 |
| Hemoptysis | 2.6766 |
| No diabetes | 2.032 |
| Nausea or vomiting | 1.6283 |
| No diarrhea | 1.5907 |
| Chronic cardiac disease | 1.5448 |
| No chronic neurologic disorder | 1.4601 |
| Chest pain | 1.252 |
| No smoking | 1.2453 |
| Rhinorrhea | 1.2076 |
| No sputum | 1.1933 |
| No myalgia | 1.1637 |
| Altered mentality | 1.1143 |
| Asthma | 1.112 |
| Diarrhea | 1.0376 |
| ADL score 1 | 1.0201 |
| No rhinorrhea | 0.9301 |
| Fatigued | 0.915 |
| Cough | 0.9135 |
| Cancer | 0.7789 |
| Chronic liver disease | 0.763 |
| Myalgia | 0.7626 |
| Sputum | 0.5087 |
| COPD | 0.4968 |
| No chronic liver disease | 0.4701 |
| Chronic neurologic disorder | 0.0798 |
| No asthma | 0.0542 |

ADL, Activities of Daily Living; COPD, Chronic obstructive pulmonary disease.

ADL score is expressed from 0 to 2, 0 being independent, 1 being partially dependent, and 2 being total dependent on others.
