# Supplementary material for: An Easy-to-Use Machine Learning Model to Predict the Prognosis of Patients With COVID-19: Retrospective Cohort Study
Source: J Med Internet Res. 2020 Nov 9;22(11):e24225. doi: 10.2196/24225 (PMC7655730; doi:10.2196/24225)
Supplement: Multimedia Appendix 3 [file jmir_v22i11e24225_app3.docx]

**Multimedia Appendix 3. Confusion matrices for the development and validation group.**

|  | **Predicted not requiring intensive care** | **Predicted requiring intensive care** | **Error** |
| --- | --- | --- | --- |
| **Derivation group** | | | |
| Not requiring intensive care | 2168 | 795 | 0.27 |
| Requiring intensive care | 37 | 295 | 0.11 |
|  | | | |
| **Validation group** | | | |
| Not requiring intensive care | 978 | 386 | 0.28 |
| Requiring intensive care | 12 | 116 | 0.09 |

Data shown for cut-off set as 0.06.
